# Surface hopping modeling of charge and energy transfer in active environments

**DOI:** 10.1039/d3cp00247k

**Published:** 2023-03-01

**Authors:** Josene M. Toldo, Mariana T. do Casal, Elizete Ventura, Silmar A. do Monte, Mario Barbatti

**Affiliations:** a Aix-Marseille University, CNRS, ICR Marseille France josene-maria.toldo@univ-amu.fr mario.barbatti@univ.amu-fr https://www.barbatti.org; b Departamento de Química, CCEN, Universidade Federal da Paraíba 58059-900 João Pessoa Brazil silmar@quimica.ufpb.br; c Institut Universitaire de France 75231 Paris France

## Abstract

An active environment is any atomic or molecular system changing a chromophore's nonadiabatic dynamics compared to the isolated molecule. The action of the environment on the chromophore occurs by changing the potential energy landscape and triggering new energy and charge flows unavailable in the vacuum. Surface hopping is a mixed quantum-classical approach whose extreme flexibility has made it the primary platform for implementing novel methodologies to investigate the nonadiabatic dynamics of a chromophore in active environments. This Perspective paper surveys the latest developments in the field, focusing on charge and energy transfer processes.

## Abstract 2

An active environment changes the photophysics of an excited chromophore. Surface hopping simulations are crucial to understanding how.

## Graphical abstract

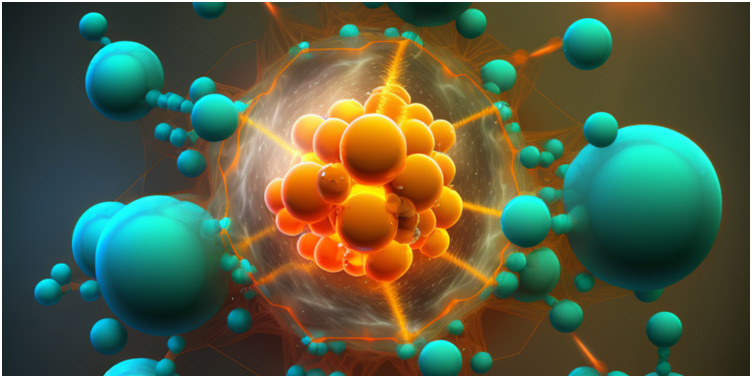

## 1. Introduction

Investigating photoinduced nonadiabatic dynamics involves understanding how the character of excited electronic states evolves, how the molecular system relaxes through the manifold of electronic states, how long this relaxation takes, how different radiative and non-radiative processes compete, and which products are formed when equilibrium is restored. As if this complexity were not enough, we climb to a new difficulty step when one must include the effect of the environment surrounding the chromophore.^1–3^ We must also consider how the environment's electronic states couple to the chromophore states, disturb their electronic density, change the potential energy surfaces topographies, dissipate the photon energy, and trigger new charge flows. Depending on the environment, excimers may be formed over multiple molecules, protons and electrons can jump to other monomers, and excitons can propagate through the supramolecular ensemble. Thus, the environment surrounding the chromophore may qualitatively change the nonadiabatic relaxation compared to an isolated excited molecule.

Consider, for example, the nonadiabatic dynamics of an isolated photoexcited nucleobase. The ground state is reached within one picosecond through a ring-puckering conical intersection.^4^ If this same nucleobase is part of a DNA strand, excimer formation involving multiple nucleobases traps the excitation for hundreds of picoseconds.^5^ Another example is pyrene. In a vacuum, it has a marked non-Kasha fluorescence due to thermal activation of the S_2_ state.^6^ This fluorescence disappears in high gas concentrations thanks to vibrational cooling to the environment. As a final example, take acceptor donor complexes at organic heterojunctions composed of thiophene oligomers (electron donors) and fullerenes (electron acceptors).^7^ The distribution of electronic states strongly depends on their relative arrangement, with on-top orientations favoring hot charge–transfer processes and on-edge orientations inducing cold charge transfer.

Many theoretical options exist for tackling the environment's effect on nonadiabatic processes. However, the surface hopping approach is likely the most popular.^8,9^ In surface hopping, the nuclei follow Newton's equations on a single Born–Oppenheimer potential energy surface (PES) at each time step, but a stochastic algorithm enables sudden switches to another surface. A swarm of many trajectories emulates the nuclear wave packet nonadiabatic evolution. Surface hopping dramatically reduces the computational costs compared to full-quantum approaches, allowing simulations of larger systems in full nuclear dimensionality and for longer periods. Moreover, surface hopping does not require PES pre-computation, making it highly flexible to simulate any system.

This Perspective overviews the intersection between nonadiabatic dynamics in active environments, charge and energy transfer, and surface hopping simulations. With hand-picked examples, we address the following questions: What does make an environment active? How does such an environment influence the nonadiabatic dynamics of a photoexcited molecular system? How can we use surface hopping to describe the charge and energy transfer between the chromophore and an active environment? Which limitations do we face? What are the emerging research focuses and future challenges?

Section 2 discusses surface hopping. As this method is well established and subject to several recent reviews,^8–11^ we briefly comment on its key aspects and limitations. Then, in Section 3, we define the features that make an environment active and establish the scope of this Perspective. Section 4 discusses how to include the environment description in surface hopping. Section 5 focuses on charge–transfer processes in their diverse aspects: electron, proton, and proton-coupled electron transfers. Section 6 tackles electronic, vibronic, and vibrational energy transfer processes. In Section 7, we take a more subjective approach to opine on which are the most promising methods and which fields deserve attention.

## 2. Surface hopping

**Fig. 1:**
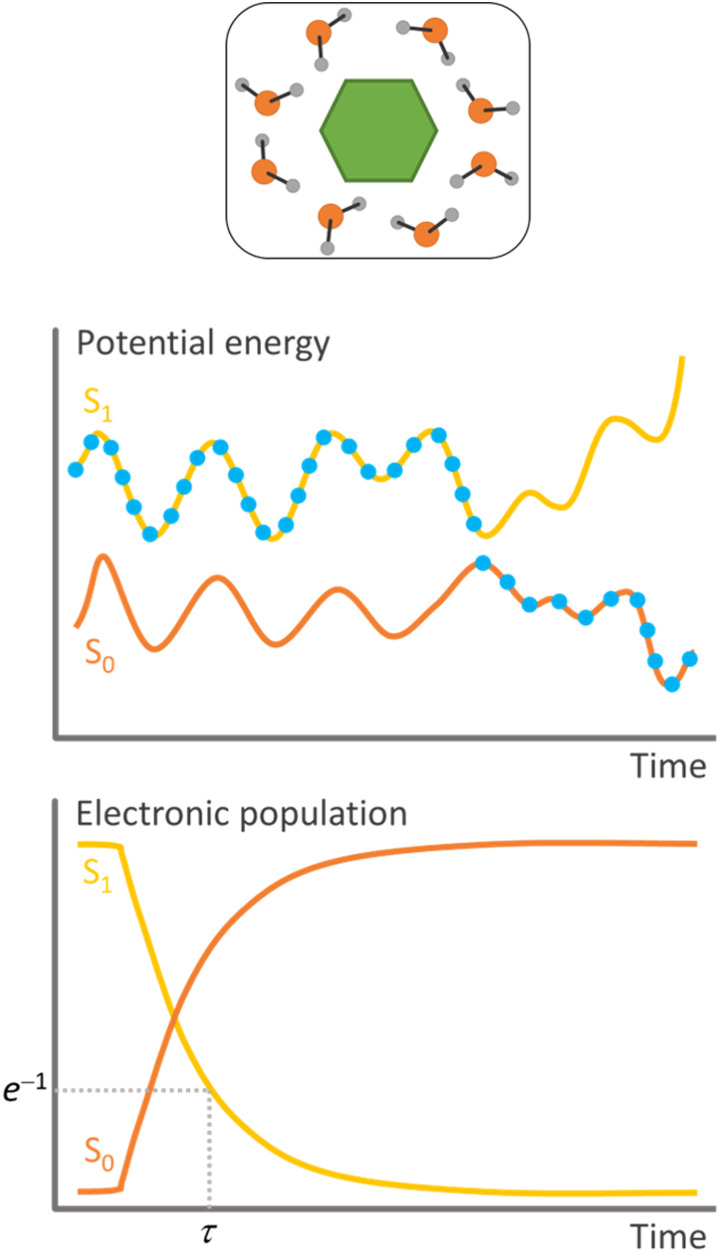
Schematic features of surface hopping. Top: Molecular system. Middle: Single trajectory representation of the potential energies as a function of time. The dots indicate the populated state at each step. Bottom: Statistics over all independent trajectories allow for estimating each state's electronic population as a function of time and determining the time constant for the internal conversion (*τ*).

Surface hopping is a nonadiabatic mixed quantum-classical dynamics method. As such, the nuclei are propagated in classical trajectories while the electrons are treated quantum-mechanically.^11^ Surface hopping uses an ensemble of independent trajectories to approximate the time propagation of a nuclear wave packet (Fig. 1). Each trajectory evolves in a single electronic state, but the electronic state can change at each integration step. The state change (or hopping) is determined by a stochastic algorithm based on the hopping probability between the current state and all other states. Whenever hopping occurs, the velocities are adjusted to conserve total energy. A trajectory may, for instance, start in the second excited state. When the nuclei reach a region of this potential energy surface coupled to the first excited state, the molecule hops to the lower state and continues evolving there. The potential energy difference between the states at the hopping time is added to the nuclear kinetic energy. Surface hopping has been extensively applied to investigate internal conversion, intersystem crossing,^12^ and photon-induced^13^ transitions in systems ranging from analytical models,^14^ through small molecules,^15^ to large supramolecular ensembles.^10^

Surface hopping is a local theory, meaning that the electronic quantities needed to propagate the equations of motion (potential energies, potential energy gradients, and nonadiabatic couplings) are computed only for the nuclear geometries of the classical trajectory. Thus, global potential energy surfaces are not required, and the electronic quantities can be evaluated on-the-fly, during the trajectory propagation. This feature makes surface hopping extremely flexible, with the literature recording simulations based on electronic structure methods ranging from time-dependent density functional tight binding (TD-DFTB)^16^ to complete active space perturbation theory to the second order (CASPT2).^17^

There are two main strategies to compute the hopping probabilities, globally or instantaneously. Global hopping probability evaluates the transition probability of a molecule after it crosses and leaves a region of nonadiabatic interactions. The Landau–Zener model^18,19^ is a well-known example of such probabilities. Nowadays, the most common global probability approaches used in surface hopping are the Zhu–Nakamura^20^ and the Belyaev–Lebedev (aka adiabatic Landau–Zener) models.^21^ The advantage of such global hopping probability approaches is that they do not require a detailed evaluation of nonadiabatic couplings. They also do not show decoherence problems we face in other variants of surface hopping, as we shall discuss. The drawback with such models is that they are derived for specific surface topographies (Landau–Zener, for instance, is derived for a linear diabatic crossing with constant diabatic coupling).^22^ Thus, it is not assured that they will perform well for some arbitrary molecular system. Another problem with global hopping probabilities is that the trajectory must be propagated until the molecule leaves the coupling region. Then, if the hopping occurs, the trajectory must be rewound to the maximum coupling point and restarted from there. This difficulty is commonly faced with a three-point strategy, which uses sequences of three timesteps to evaluate the global hopping probability.^23^

On the other hand, instantaneous hopping probabilities are, as the name implies, defined for each time step. The most famous example of this strategy is the fewest switches surface hopping, FSSH.^14^ This approach propagates a local approximation of the electronic time-dependent Schrödinger equation. Then, this equation's electronic coefficients are used to predict the hopping probability. FSSH does not make any assumption on the topography of the potential energy surfaces. Nevertheless, it has a significant shortcoming related to an excess of coherence.^24^ The nondiagonal terms of the density matrix formed with the electronic coefficients do not drop to zero as fast as in full quantum systems, a problem that stems from the independent-trajectory approximation.^25^ This over-coherence is faced using different strategies to force decoherence.^26^ We will discuss decoherence further in the context of electron transfer (Section 5.1). FSSH needs nonadiabatic coupling vectors to integrate the time-dependent Schrödinger equation. Nevertheless, this requirement can be alleviated using time-derivative couplings computed from wave function overlaps^27^ or energy gaps in the time-dependent Baeck–An approach.^28^

No matter the specific approach, surface hopping will have problems with non-local nuclear effects such as tunneling and quantum interferences.^29^ Moreover, as with any method based on classical trajectories, zero-point energy leakage from high-frequency vibrational modes may be a problem, especially in systems composed of multiple non-bonded units.^30^ Trivial crossings (crossings between electronic states with little overlap between them) in extended systems may also cause troubles during the simulation,^31,32^ requiring special techniques such as local diabatization^33^ or flexible surface hopping.^34^

Surface hopping simulations based on methods without nonadiabatic coupling vectors may suffer from a size-extensivity problem due to excess kinetic energy artificially enabling back hoppings (hoppings to upper states). Large systems, like those including the environment, may be especially prone to this problem, which can be addressed with different techniques.^6,35–37^

In general, conventional surface hopping will perform well if (1) the light pulse is shorter than the excited-state dynamics, (2) the nuclei move fast like quasi-classical particles, (3) there are no significant recoherences between nuclear wave packets, and (4) non-local effects can be neglected. However, even if these conditions are not satisfied, specific surface hopping implementations may be available to extend its validity domain, for instance, including fast nuclear degrees of freedom in the quantum partition^38^ or explicitly accounting for the electromagnetic field in the Hamiltonian.^39^ We will survey many of these surface hopping variants within the following sections.

### 2.1 Exact factorization

Exact factorization^40^ has been proposed as an alternative to the conventional Born–Huang expansion of the molecular wave function. Exact factorization is still in its early days and is not a routine methodology to deal with active environments. We mention it here primarily due to its potential to create new ways of simulating the nonadiabatic dynamics of these systems.

In the exact factorization representation, the time-dependent Schrödinger equation is translated into two coupled differential equations driven by time-dependent scalar and vector potentials.^41^ What initially seems a complication offers an outstanding new manner to propagate the nuclear dynamics. In nonadiabatic dynamics based on the Born–Oppenheimer approach (standard surface hopping included), the molecule evolves through multiple time-independent potential energy surfaces, with the transition between them mediated by nonadiabatic couplings. Conversely, a single time-dependent potential energy surface dictates the nuclear dynamics in exact factorization.^42^

Algorithms employing an approximate trajectory-based description of the nuclei in the framework of exact factorization have been developed, such as the coupled-trajectory mixed quantum-classical (CT-MQC) dynamics. Furthermore, different surface-hopping approaches based on exact factorization have been proposed.^43–45^

In the context of proton-coupled electron transfer, the first exact-factorization model appeared in 2019 when Agostini *et al.* applied it to simulate the dynamics of a 1D model.^41^ Recent works have set the first steps toward more realistic systems,^46,47^ with approaches that can be potentially extended to address environment and tunneling.

## 3. Defining an active environment

### 3.1 Definition and scope

For this Perspective, we define environment as any atomic or molecular systems surrounding the chromophore where the electronic excitation is not initially spread over. These systems may be composed of other molecules, extended materials, or even covalently bound to the chromophore. Consider the example we discussed in the introduction, a nucleobase in a DNA strand. The nucleobase is the chromophore absorbing UV radiation, while the rest of the strand, including the immediately attached sugar group, is the environment.

**Fig. 2:**
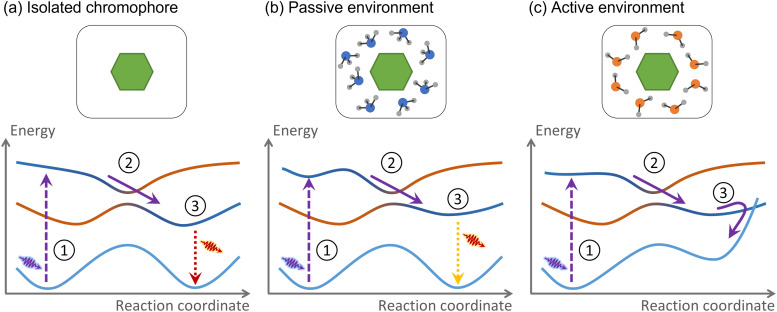
We define an active environment according to its effect on the photodynamics of the chromophore. (a) The hypothetical chromophore is excited (1), relaxes to the first excited state (2), and fluoresces (3). (b) A passive environment does not qualitatively change the photodynamics compared to the isolated case. (c) An active environment induces entirely new photodynamics.

The environment is active if nonadiabatic dynamics following the chromophore photoexcitation diverges from that of the chromophore in a vacuum (Fig. 2). As we mentioned, the nonadiabatic relaxations of a nucleobase in a vacuum and DNA are distinct. Therefore, DNA is an active environment. Alternatively, suppose the nucleobase is solvated within a water–methanol solution.^48^ The internal conversion time constant may be affected, but the nonadiabatic mechanism remains the same.^49^ Thus, the water–methanol solution in this specific example is passive.

As we defined, an active environment is a broad concept and can be used in various contexts, ranging from atmospheric aerosols,^50^ multichromophoric systems,^51,52^ and photoactive proteins^53,54^ to molecular crystals,^55^ solid-state organic materials,^56^ and metallic surfaces.^57^ It helps to focus beyond all this complexity to think that the effect of the active environment can be classified into three types: changes in the PES topography, energy transfer, and charge transfer between the chromophore and the environment. Moreover, confined electromagnetic fields can also act as an active environment.^58^

This Perspective will focus on active environments involving charge and energy transfer only. Nevertheless, before diving into them, the following two subsections briefly survey the other two types, active environments involving PES distortions and confined electromagnetic fields.

### 3.2 Environment-induced PES topography distortion

When we state that the action of the environment occurs through three different effects—changes in the PES topography, charge transfer, and energy transfer—there is a degree of ambiguity we want to elucidate before continuing. All three translate into PES distortions. However, this subsection only concerns PES distortions that do not imply a charge transfer and that the nonadiabatic process occurs faster than any energy flow between the chromophore and the environment. If these conditions are satisfied, we say the environment is perturbative (if they are not satisfied, it characterizes *an intrusive* environment).

A perturbative environment can still be active. The topographic distortions they cause can profoundly change the nonadiabatic processes. A well-known example is the effect of a protic solvent on an organic molecule with exposed lone-pair electrons. Take 2-aminopurine (2AP), for instance. In the gas phase, photoexcited 2AP returns to the ground state *via* internal conversion. In water, however, it is fluorescent.^59^ The reason is the strong stabilization of the nπ* state (compared to the ππ*) induced by a hydrogen bond between 2AP and water.^60^

Another common type of perturbative environment inducing new nonadiabatic dynamics is cage effects. In this case, the chromophore is trapped by weak interactions (van der Waals, hydrophobic, π–π interactions, and hydrogen bonds) within a molecular cavity, a crystal, or even encapsulated by solvent molecules.^61^ For instance, photo-dimerization of stilbene leading to cyclobutane can be totally restrained using cyclodextrin as a host.^62^

Nonadiabatic dynamics in a perturbative environment are more straightforward to simulate than in those where charge and energy transfers play a role. In principle, we can simulate chromophores in such environments with standard methods like surface hopping based on conventional QM/MM^63^ or even continuum models in some specific cases.^64^ Nevertheless, these simulations still require a good tuning of the electronic structure level to be used together with surface hopping. Ref. 65 delivers an excellent introduction to the accuracy of using different solvation models in an organic chromophore. The advantages and limitations of using continuum solvation methods and atomistic descriptions of the solvent are discussed in ref. 66 and 67 (see also Section 4.1).

Surface hopping with QM/MM (see Section 4.2) has been the workhorse for simulations of environment-induced PES distortions. Among many examples,^53,54,68^ it was used to reveal how an argon matrix prevents the dissociation of trapped formamide, leading to the formation of weak-interacting complexes.^69^ It predicted that the nonadiabatic dynamics of a tetracyanoethylene/anthracene complex in the gas phase and water are entirely distinct.^70^ It helped understand why 4-(*N*,*N*-dimethylamino)benzonitrile (DMABN) exhibits a single fluorescence band in the gas phase but dual fluorescence in polar environments.^71^ The recent simulations by Palombo and co-authors illustrate the state of the art of such a QM/MM surface hopping approach.^63^ In that work, they employed it to explain the fluorescence of the retinal chromophore within neorhodopsin. This group has also developed protocols for automatically generating QM/MM models.^72^

### 3.3 Confined electromagnetic-field environment

Although it goes beyond the scope of this Perspective, restricted to atomic and molecular environments, we cannot avoid mentioning that the nonadiabatic dynamics of an excited chromophore can also be affected by confined electromagnetic fields in the so-called polaritonic chemistry.^73,74^ Over the last years, polaritonic chemistry has emerged as a gateway for remarkable functionalities but imposing a plethora of theoretical challenges. Polaritons are hybrids between light and matter and occur when the light-matter interaction becomes strong enough.^75^ This strong coupling regime can significantly modify the photochemistry and photophysics of molecular systems, opening novel opportunities to control chemical transformations.^74,76^ Once more, surface hopping is up to the challenge.^77^

In a recent publication, Fregoni *et al.* present theoretical strategies and challenges in polaritonic chemistry, mainly focusing on situations where molecular electronic transitions are coupled to light modes.^73^ In this context, surface hopping is a powerful approach to describe the system's dynamics. Besides being less demanding than quantum dynamics-based methods, surface hopping can describe many nuclear degrees of freedom and cavity losses *via* quantum jump algorithms. Another advantage is the facility to include an atomistic description of the solvent *via* QM/MM electrostatic embedding. The significant deficiencies of surface hopping are the incapacity of describing potential tunnelings and the inaccurate evaluation of transition probabilities in the presence of several quasi-degenerate states,^24^ typically observed when many molecules couple to a single cavity mode.^73^

Fregoni and co-workers extended surface hopping to investigate photochemistry in the strong coupling regime and applied it to manipulate azobenzene isomerization.^77,78^ The critical points are to mimic a plasmonic nanocavity and consider both the photon degrees of freedom and nonadiabatic events in the dynamical description.^78^ In the case study reported in ref. 39, azobenzone (treated at a semiempirical level) interacted with the MM environment composed of an organic cage and explicit water molecules inserted between gold layers. Another example is in the work of Antoniou *et al.*, who performed surface hopping on surfaces derived from their non-Hermitian formalism to model vibrational relaxation.^79^ The effect of cavity loss was considered through the Langevin approach, including a random force and a frictional term to mimic vibrational relaxation. In principle, such cavity loss effect could also be achieved *via* surface hopping on complex-valued potential energy surfaces.^80^

## 4. Computational strategies to simulate active environments

**Fig. 3:**
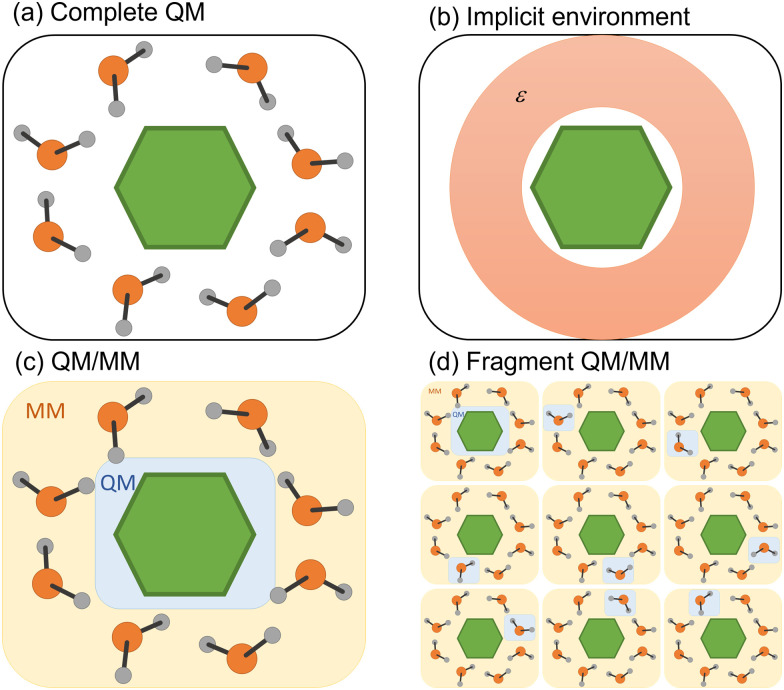
Four different strategies to simulate the environment surrounding the chromophore. (a) QM treatment of the entire system. (b) Implicit solvation. (c) Hybrid QM/MM. (d) Fragment QM/MM approach.

Any attempt to include the environment's effect on a simulation often carries the heavy burden of increasing the computational cost. Different strategies to decrease this cost have been deployed, whether considering the environmental effect in terms of its macroscopic properties or explicitly including solvent molecules in the simulation (Fig. 3). We discuss both strategies in the following subsections.

### 4.1 Implicit environment

The simplest way to include solvent effects is by using implicit solvation (Fig. 3b). In this approach, the environment is represented by an electrostatic field where no specific solute–solvent interactions are considered.^81^ The solute is confined to a cavity, and a dielectric medium characterizes the surrounding environment.^82^ The mutual solvent–solute polarization alters the charge distribution, reflecting within the final dipole moment of the solute. Two dynamical regimes emerge within standard continuum model implementations: non-equilibrium and equilibrium. In the first case, the solvent's electronic polarization (*i.e.*, the fast degrees of freedom) is instantaneously equilibrated with the state of interest, while its inertial degrees of freedom (*i.e.*, the slow ones) are still equilibrated with the initial state. In the equilibrium regime, all degrees of freedom of the solvent are instantaneously equilibrated with the electronic density of the state of interest, which comes with an additional computational cost.^67^

However, modeling nonadiabatic dynamics using continuum solvation may suffer from conceptual problems concerning solvation dynamics.^83^ Upon photoexcitation, the solute's electron density change induces a solvent response. Continuum models assume that this response is not immediate; thus, the solvent remains equilibrated with one electronic state of the solute.^66^ However, in nonadiabatic dynamics, the entire system is out of equilibrium, leading to an unreal instantaneous solvent polarization. Moreover, non-linear problems can arise from the solvent reaction field, which can induce an additional time-dependent coupling between different excited states.^67^

Nevertheless, implicit solvation may still be a reasonable strategy when no specific solute–solvent interactions affect photophysics, for example, relaxation upon ultrafast time scales.^67,84^ For instance, Hammes–Schiffer and co-workers showed that modeling electron transfer reactions using surface hopping with implicit models was a valid option even for solvents exhibiting complex relaxation behavior with multiple relaxation time scales.^85^ Ref. 67 and 86 discuss equilibrium and non-equilibrium regimes in nonadiabatic dynamics more extensively. In particular, Spezia *et al.*^86^ showed that surface hopping results might significantly differ between the two cases. Nonetheless, the equilibrium solvation description can still provide a good description of a crossing seam resulting from chromophore–solvent interaction.

### 4.2 Explicit environment

In many situations, the photophysics of the system is affected by specific solute–solvent or non-electrostatic interactions (dispersion and Pauli repulsion) that require explicit solvent molecules.^87^ These situations are common in systems where the chromophore interacts with the solvent through hydrogen bonds or π-stacking.^88,89^ Suppose the interaction is local, and only a few molecules are necessary around a specific substituent. In that case, all molecules could be treated quantum-mechanically using a cluster model.^90,91^ In other cases, the global effect cannot be mimicked by well-positioned solvent molecules, but rather the entire bulk effect must be simulated. Alternatively, one may want to investigate the energy transfer between the chromophore and the solvent.^88^ In either case, treating all molecules quantum-mechanically is unfeasible. A commonly employed strategy is to split the complete system into quantum-mechanical (QM) and molecular-mechanical (MM) parts, running the surface hopping simulations with a QM/MM hybrid approach (Fig. 3c).^92^

Most QM/MM surface hopping studies work within an electrostatic embedding where the QM region explicitly feels the electrostatic interactions of the point charges composing the MM region. However, these MM point charges are still those of the ground-state electronic configuration, and any nonelectrostatic interaction or mutual polarization between QM and MM parts is entirely neglected.

QM/MM formulations allowing mutual polarization between the MM and QM parts have been developed^68,83,93–95^ and, more recently, used with surface hopping.^96^ Nonetheless, the combination of nonadiabatic dynamics with polarizable QM/MM suffers from inherent difficulties due to the dependence of the polarization degrees of freedom of the environment on the QM charge density.^96^ This introduces a nonlinearity in the system's Hamiltonian and, as a consequence, different responses of the polarizable embedding to different excited states. A strategy is to use a linear-response scheme, which can address multiple states simultaneously but lacks the contribution induced by changes in the electronic density when moving between electronic states. Conversely, a state-specific approach would account for polarization changes induced by hopping between excited states, but this scheme cannot deal with multiple excited states at the same time. The better performance of state-specific framework is shown in ref. 96. Strategies to include polarizable environment into nonadiabatic dynamics are discussed in ref. 83.

Another significant limitation of conventional QM/MM is its inability to exchange particles between the QM and MM regions (during a proton transfer, for instance) and to transfer electronic excitations from the QM to the MM regions (such as in exciton transport). The first problem can be addressed using adaptive QM/MM, which dynamically defines the QM and MM partitions.^97^ The second problem can be alleviated with two different types of divide-and-conquer strategies based on fragment approaches. Both help to treat multichromophoric systems where the QM part needs to be enlarged.

In the first strategy, the electronic state of the molecular system at each time step is built from permutating QM/MM calculations of each monomer in the MM field of the others (Fig. 3d).^98^ Such an approach (dubbed EXASH for exciton approach for surface hopping) is still limited to Frenkel exciton Hamiltonians, not allowing the description of charge transfer between units. However, at least partially, it can account for environment polarization through the excited-state charges used to compute exciton couplings.

The second strategy employs fragment molecular orbital approaches (FMO)^99^ to compose a supramolecular wave function from molecular orbitals at each molecular unity, allowing the charge and energy transport between sites.^100–102^ This concept was explored in the fragment orbital-based surface hopping (FOB-SH),^100^ in which a diabatic electronic Hamiltonian is parameterized through force fields and FMO intersite overlap matrices. Then it defines a one-particle time-dependent supramolecular electronic wave function for the charge excess as a linear combination of molecular orbitals strongly localized on a single fragment.^100^ These orbitals are built from single-occupied Kohn–Sham orbitals obtained for the isolated fragment. A locally-approximated Schrödinger equation is used in an FSSH algorithm to allow the charge to jump between sites (but always in the same adiabatic surface). The classical forces acting on the nuclei are computed from the force field. Under the name of Frenkel exciton surface hopping (FE-SH), this FMO approach has also been formulated to allow localized exciton transport.^103^ It has also been generalized to simultaneously address charge transport, exciton transport, and electron–phonon coupling in the excitonic state-based surface hopping (X-SH).^104^ In all these methods, electronic structure calculations are only carried out to parameterize the diabatic Hamiltonian matrix elements but not during the dynamics, making them reasonably inexpensive.

Treatment of the entire molecular system at the QM level (we may call it a brute force strategy; Fig. 3a) is feasible at the cost of applying strong approximations. For instance, Wang, Akimov, and Prezhdo have developed a computational protocol that enables surface hopping for extended systems with hundreds of atoms treated at the QM level.^10^ This protocol has two main features. First, excited states are treated as single Kohn–Sham determinants within a time-dependent approach (TD-KS). Second, trajectories are propagated in the ground state, while hoppings are evaluated between excited states. The rationale for this approximation, named neglect of back reaction (NBR, aka classical reaction path), is that in an extended, rigid system, the nuclear distortions due to the excited state dynamics can be neglected. Surface hopping based on NBR has often been used to simulate nonadiabatic dynamics in nanoscale materials.^105^

## 5. Charge transfer

**Fig. 4:**
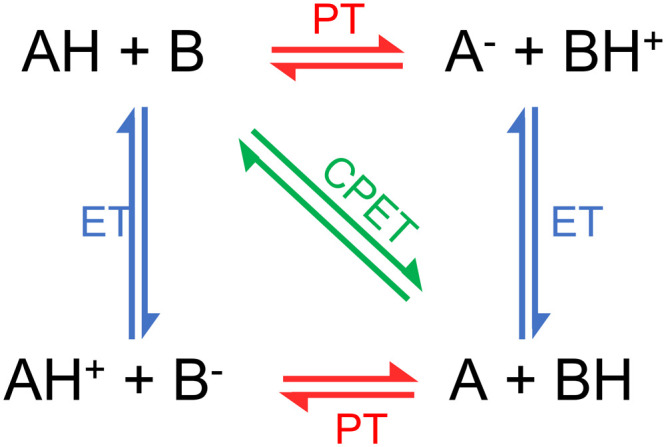
Charge transfer processes: proton transfer (PT), electron transfer (ET), and proton-coupled electron transfer (PCET). PCET can be concerted (CPET) or sequential (either ET-PT or PT-ET). Figure adapted from ref. 106.

We usually refer to either electron or proton transfer when we speak of charge transfer in a molecular system. It is also possible to have coupled proton–electron transfer processes. Fig. 4 schematically summarizes all these charge–transfer processes. In the following subsections, we discuss how surface hopping has been applied to investigate each of them.

### 5.1 Electron transfer

Electron transfer is at the center of many biological phenomena, such as respiration,^107^ photosynthesis,^108^ damage and repair of DNA,^109^ and magnetic compass in birds,^110^ but also in technological applications, including polymer light-emitting diodes, photovoltaic devices, and organic field-effect transistors.^111^ An increasing interest in simulating electron transfer in metal and semiconductor surfaces and interfaces has emerged in the last few years, as they are crucial for molecular junctions,^112–115^ dissociative chemisorption,^116–119^ photocatalysis, solar cells, waste processing, and quantum confinement devices.^120–124^ Several perspectives on nonadiabatic dynamics and theoretical aspects of charge transport are available.^125–127^ We refer the interested reader to ref. 128 and 129 for more details on electron transfer theory.

#### 5.1.1 Electron transfer with surface hopping

Consider a supramolecular system where electron transfer between units may occur. A conventional rate approach, for example, using *Marcus theory* or the *Bixon–Jortner model*,^111^ requires knowing the nuclear reaction coordinates inducing the transfer process and making assumptions on the initial and final diabatic states. Reaction coordinates, however, are not evident in extended systems, and several restrictive assumptions underlie those theories (such as neglecting spatial delocalization of charge carriers and excitons), which can strongly affect exciton dissociation and charge transport efficiency.^104,130^ Dynamics simulations in full dimensionality, as in surface hopping, excel in this regard. They naturally reveal the reaction coordinates without imposing any underlying assumptions about the nature of the electron transfer (adiabatic *vs.* nonadiabatic) and the degree of hole localization.

Take, for instance, the surface hopping simulations of adenine microsolvated in a water cluster reported in ref. 131. The 9H isomer behaved similarly to isolated adenine, with the molecule returning to the ground state through a ring-puckering state intersection. Thus, the water cluster was a passive environment for 9H-adenine. Nevertheless, the simulations of 7H-adenine revealed a completely unexpected nonadiabatic pathway induced by an electron transfer from one of the water molecules to adenine. Surface hopping became an invaluable tool for understanding electron transfer in the most diverse systems thanks to its high discovery power. It, however, faces the challenge of treating the extended supramolecular system at a quantum mechanical level.

Combining surface hopping with time-dependent density–functional tight biding (TD-DFTB) is an alternative to reduce the computational cost of electronic structure calculations.^132^ This is possible due to a semiempirical approximation to DFT used in DFTB. Extensions of the method were proposed and combined with surface hopping. For instance, Darghouth *et al.* use TD-lc-DFTB, a version that includes long-range corrections (lc), to investigate charge and energy transfer in an organic solar cell heterojunction consisting of a pentacene molecule on the top of a buckminsterfullerene.^133^ In this work, they tested several values for the range-separation parameter (*R*_lc_) and extracted electron transfer and charge transfer times as a function of *R*_lc_ using a kinetic model. They could predict charge transfer at hundreds of femtoseconds and have initial physical insights into processes happening at longer time scales, such as exciton diffusion and charge separation. According to the authors, their model should not be seen as quantitative but rather as a step towards a more realistic modeling of charge transfer at organic heterojunctions.

A workhorse protocol for electron-transfer simulations has been FSSH based on TD-KS and NBR approximations discussed in Section 4. In this strategy, no parameterization is needed. Kilina *et al*. applied FSSH based on TD-KS to investigate the relaxation of charge carriers in quantum dots.^134,135^ Since then, this methodology has been used in several condensed-phase materials.^125,128,136–141^ Kang and Wang incorporated NBR approximation and decoherence correction within a density matrix approach to nonadiabatic dynamics.^142^ More recently, Smith *et al*.^143^ extended the NBR approximation to include many-body effects. They showed that these effects accelerate the nonradiative decay in nanocrystals by a factor of 2–4 compared to a single-particle picture.^143^

In a recent publication, Shakiba *et al.* reported a new methodology that uses NBR approximation and Grimme's extended tight-binding (xTB) parameterization to investigate nonadiabatic dynamics in periodic systems up to 1500 atoms.^144^ They demonstrate the reliability of this approach by modeling “hot” electron relaxation dynamics in a large silicon nanocrystal and electron–hole recombination in a titanium-based metal–organic framework and graphitic carbon nitrite monolayer. The xTB approach yielded nonadiabatic couplings and overall dynamics comparable to DFT/PBE level for the three systems. In that work, they also proposed an improved scheme for computing nonadiabatic couplings between pairs of excited states, which utilizes the Libint2 library for the analytical computation of time-overlap integrals. They tested various surface hopping and decoherence schemes in the framework of xTB.

Fragment approaches (see Section 4.2) have also played a core role in enabling surface hopping simulations of electron transfer in extended systems. Akimov developed a trajectory-based approach that combines FMO and tight-binding extended Hückel theory for modeling charge and energy transfer in large systems.^101^ The critical element is the reduced number of molecular orbitals used from each fragment. The NBR approximation was adopted, with the ground state nuclear dynamics computed using classical force fields, and the time-dependent FMOs were determined from the geometries of each fragment along this trajectory. The methodology is expanded using Markov-state surface hopping. The linear scaling performance of the method allows modeling long-timescale charge transfer in systems with hundreds of atoms. The procedure was validated in the charge transfer investigation in subphtalocyanine/C_60_ heterojunction.

Giannini and Blumberger proposed a fragmented approach named FOB-SH (Section 4.2), primarily designed to model charge transport in organic crystals^130^ and biological macromolecules.^145^ The coarse-grained description of the material's electronic structure makes this method computationally efficient. It uses a tight-binding electronic Hamiltonian with matrix elements parameterized to explicit electronic structure calculations, which is updated on-the-fly along the trajectories. The charge carriers may be localized in any fragment unit, and their energies are computed at the semi-empirical level using the force field of correspondingly-charged isolated molecules. The strength of the method is the accurate calculation of charge carrier mobilities in organic crystals.^146^ Using FOB-SH in future applications may provide fruitful insights about charge transfer in 2D covalent organic framework materials.^130^

An extension of FOB-SH, named X-SH, has been developed to simulate exciton dissociation to charge carriers in optoelectronic materials at the nanoscale.^104^ It combines elements from FOB-SH and FE-SH, employing a diabatic Frenkel exciton Hamiltonian that includes charge–transfer, exciton-transfer, and electron–phonon-coupling terms. X-SH was tested for a 1D model of a fullerene–oligothiophene interface, predicting the modes which receive the excess energy, decay dynamics, and state population at ultrafast timescale, providing results comparable to quantum wave packet propagation.^104^ However, as FOB-SH, X-SH does not include nuclear quantum effects (such as nuclear tunneling and zero point energy), which may be necessary for studying charge and exciton transport, particularly at low temperatures.

Ghosh *et al.* combined FOB-SH and ring polymer surface hopping (RPSH) to account for nuclear quantum effects. They applied these methods to a molecular dimer model and investigated the hole transfer rate's dependence on temperature and driving force.^147^ Among the three flavors proposed, *i.e.*, bead approximation (RPSH-BA^148^), weighted bead approximation (RPSH-wBA), and isomorphic Hamiltonian method (SH-RP-iso^149^), they found that the latter is the most promising for including zero-point motion and tunneling in charge transport simulations in biological systems and molecular materials. However, although this method can improve the description of nuclear density in surface hopping, it still suffers from other limitations of traditional surface hopping, such as over-coherence.

Nonadiabatic dynamics does not impose assumptions about localization or delocalization of the excess charge. However, different electronic representations and propagation schemes could lead to a greater or lower degree of charge delocalization.^150,151^ For instance, charge delocalization only occurs near regions of large nonadiabatic coupling in surface hopping. On the other hand, the system is expected to be in a superposition of states, and the charge will be delocalized in more fragments in the mean-field methods. The environment's response to this charge will be different depending on the method.^150,151^ To address this problem, Kubař and Elstner studied the various degrees of charge delocalization in DNA oligonucleotides obtained with surface hopping and mean-field Ehrenfest methods.^102^ Simulation of a DNA oligonucleotide requires a significant computational cost reduction due to the system's size. For that reason, Kubař and Elstner used a linear combination of fragment orbitals with DFTB. The solvent's interactions were included with QM/MM and a non-polarizable force field, corrected with a scaling factor. In their surface hopping implementation, they did not rescale the velocities after hopping to ensure energy conservation, did not treat classically forbidden transitions, nor applied decoherence corrections. Despite those approximations, they could follow how each nucleobase's occupation changes over time.

#### 5.1.2 Impact of decoherence corrections on electron transfer

There has been some debate in the literature regarding including decoherence effects to recover the correct scaling of the electronic coupling in electron-transfer rates obtained with FSSH. Landry and Subotnik showed that the FSSH rates without the decoherence correction scale linearly with the diabatic coupling instead of an expected quadratic dependence.^152^ Since then, several authors^34,153–155^ have addressed the decoherence problem, leading to apparent contradictions in the literature.

Jain and Subotnik conducted a systematic study with a wide range of parameters considering rates obtained directly from the population decay and transition state theory.^154,156^ They showed that this theory and population decay results only agree after including decoherence corrections. FSSH without decoherence predicts incorrect rates for highly exothermic electron transfer. Nevertheless, it reasonably predicts lifetimes and rates for isoenergetic electron transfer.^154^ They conclude that the results depend not only on including decoherence corrections but also on the decoherence method. Smith and Akimov also discuss decoherence effects and methods in recent surface hopping applications for modeling charge transfer in condensed matter materials.^105^ In cases like tight-binding FMO-based dynamics,^101^ Akimov found that including decoherence is critical to predicting reasonable CT time scales in their interfacial heterojunction model.

A benchmark with different coherence-corrected methods in donor-bridge-acceptor models highlights the importance of carefully choosing the decoherence–correction method.^157^ While augmented FSSH (A-FSSH) quantitatively recovers Marcus theory within the superexchange regime, the decay-of-mixing approach^158^ only delivers a qualitative agreement. Moreover, a partial inclusion of nuclear quantum effects in A-FSSH within Marcus theory was developed.^159^ Still, one must deal with the challenges of accounting for the quantum and environmental impact, the prohibitive computational cost for long-time-scale electron transfer, and extracting information about the electron-transfer rate from the available observables after the simulation (see Section 5.1.3).

#### 5.1.3 How to monitor electron transfer during dynamics

**Fig. 5:**
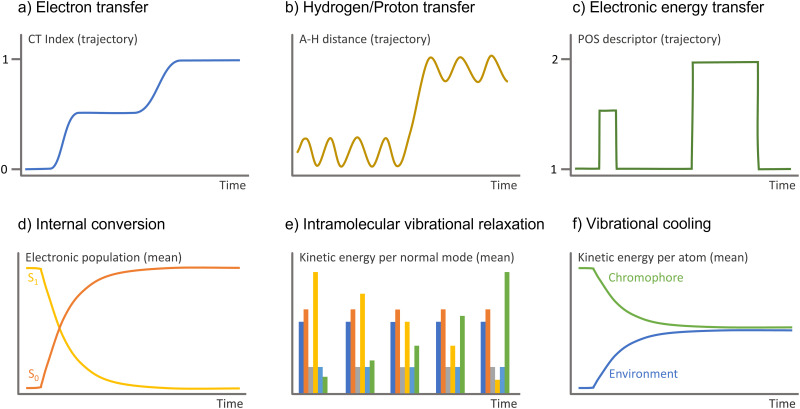
Observable to monitor in the surface hopping results to identify diverse charge and energy transfer processes. A–H is the interatomic distance between the hydrogen and the atom it attaches.

How can we define an electron-transfer event in surface hopping? A crucial step is determining the relevant observables. We will find a few different approaches in recent literature. Cardozo *et al*. used a density-matrix-based method to estimate the charge transfer in the dynamics of the benzene excimer.^160^ At each time step, they calculated a charge–transfer (CT) index and the exciton's average position^161^ based on the partition of the transition density matrix. Such an approach is particularly suited for surface hopping because it can be done after the simulations are finished, post-processing a random sampling of snapshots to compute the density descriptors, producing a result as that illustrated in Fig. 5a.

CT indexes were also used to investigate the role of triplet states in the ultrafast dynamics of azurin sensitized with a Re complex in water, including implicit solvent effects.^162^ In a similar fashion, Liu *et al*. estimated electron transfer in FSSH simulations of the interface between ZnPc and MoS_2_.^163^ They obtained the total electron charge of (pre-defined) fragments by summing the contribution of all basis functions belonging to the atoms of those fragments, similar to a Mulliken population analysis. In this way, it was possible to follow the amount of charge transferred between the fragments through the simulation.

Akimov *et al*. estimated the electron transfer rates following the population decay of the density of states.^164^ They studied the photoinduced electron-transfer mechanism in N-Ta_2_O_5_ sensitized with Ru complexes, which is vital for photocatalytic CO_2_ reduction. They used information from the projected density of states to identify the relevant donor and acceptor states localized on an N-Ta_2_O_5_ surface and Ru complexes and followed the population decay of those states.

Alternatively, an electron-transfer event can also be monitored by the number of transitions between the final and initial adiabatic states per unit of time. In the transition count rate strategy, a transition between the donor and the acceptor is counted when the population changes within a given threshold (for instance, from greater than 0.9 to less than 0.1), which can sometimes be very complex.^102^ Any comparisons with electron-transfer theory should be made carefully since this threshold is arbitrary.^165^

Population decay and the number of transitions lead to the same result if the electron-transfer event can be treated as a point charge transferred from the donor to the acceptor.^165^ Charges are treated quantum-mechanically in surface hopping, possibly leading to charge delocalization over multiple units. This effect is accounted for only in the population decay method. Indeed, electron-transfer rates derived from the number of transitions and population decay methods agree with Marcus theory in the small coupling regime (≤*λ*/10, where *λ* is the reorganization energy).^165^ The number of transitions method agrees with Marcus theory for a wide range of *λ*, arguably due to a cancellation of errors. (Note that Marcus theory considers that electron transfer involves a direct transfer from the donor to the acceptor state, neglecting charge delocalization.) Population decay rates have a good agreement with Marcus theory for small *λ* values but deviate by a factor of 5 for larger values.^165^ Therefore, the number of transitions method is more suitable if the results are compared to Marcus theory. On the other hand, the population decay method is more suitable for comparisons with experimental population decay measurements.^166^

Recently, Titov *et al*.^167^ highlighted that one could achieve different conclusions regarding charge delocalization depending on how the ensemble of trajectories is analyzed in surface hopping. To address this problem, they used the fractions of transition density matrices (FTDMs) method to evaluate the exciton delocalization in a tetracene dimer. The FTDM descriptors were computed considering two average schemes: one that divides the dimer into left and right fragments, and an average FTDM is computed for each one (*i.e.*, FTDM_L_ and FTDM_R_); and a second scheme where the highest FTDM and lowest FTDM are defined at each time step and, subsequently, one computes an average FTDM_H_ and an average FDTM_L_. Their results show that while the left-right FTDM method predicts a delocalization of the exciton, the highest-lowest method predicts exciton localization, indicating that the interpretation of the results in surface hopping depends on the quantity averaged.

### 5.2 Proton transfer

Proton transfer is a fundamental process in chemistry and biochemistry. It is linked to the Brønsted concept of acid–base reactions and describes acid–base catalysis in solution, proton channels, and proton conduction in water, among other processes.^168^ This process can occur intra- or intermolecularly in the ground or excited states. However, solvent effects can markedly impact proton transfer.^67^ One of the most common examples is the excited-state intramolecular proton transfer (ESIPT) reactions.^169^ Further complications arise when ESIPT competes or is coupled with charge–transfer reactions, as discussed in ref. 170. Other examples are photoacids and photobases chromophores, which experience dramatic changes in the p*K*_a_ upon excitation, inducing proton transfer reactions with the solvent.^171^

#### 5.2.1 Proton transfer with surface hopping

Surface hopping applied to proton transfer in solution has been restricted to cases where the transferred proton is in the QM region, either in inter- or intramolecular proton transfer. The reason for such imposition is simple: such a phenomenon involves the break and formation of chemical bonds. For example, in a recent application of surface hopping to proton transfer in water, the intra- and intermolecular excited-state proton transfer of 3-hydroxyflavon (3-HF) were studied.^172^ The goal was to explain its experimentally observed dual fluorescence in solvents containing protic contamination (water) *vs.* the single fluorescence band in highly purified nonpolar solvents. The modeling consisted of a cluster formed by 3-HF and 1 up to 5 water molecules immersed in the bulk solvent described with a continuum model. For the isolated 3-HF, ultrafast ESIPT from the enol group to the neighboring keto group was observed, with the proton being transferred with a time constant in good agreement with the experimental value obtained in a nonpolar solvent. Adding one water molecule quenched this intramolecular transfer process, which was replaced by an excited-state intermolecular proton transfer *via* the bridging water molecule. Adding more water molecules leads to significant inhibition of this intermolecular proton transfer. The initial excited-state enol structure is highly preserved in the dynamics, an outcome pointed as the origin of the violet–blue fluorescence appearing in the solvents contaminated with protic components.

A way to consider bulk effects is by including a continuum model around a small cluster formed by the chromophore and well-positioned solvent molecules. Alternatively, the bulk solvent can be represented as MM molecules. Moreover, in the case of proton transfer, both the proton donor and acceptor must be included in the QM region. One advantage of simulating the bulk solvent by this hybrid QM/MM approach is that the solute–solvent and solvent–solvent collisions are reasonably well described, allowing to address phenomena such as vibrational cooling.^88^

Simulations of extended systems where the proton transfer involves molecules distant from the chromophore are still challenging for surface hopping. Consider, for instance, the transfer occurring across an extended chain of water molecules (Grotthuss mechanism).^173^ In such a case, one needs to include this chain of the solvent molecules in the QM region, which can be computationally very demanding. Even if we have a reasonable number of molecules in the QM region, some of these solvent molecules may switch places with another from the MM region during the dynamics, artificially inhibiting the proton transfer. A way to cope with this problem is employing an adaptive QM/MM, in which the description of the migrating species needs to be updated as they diffuse away.^97,174^ Even for this approach, describing the boundaries between the QM and MM regions is arduous and can strongly affect the proton-transfer rates.^174^ Nevertheless, for a more accurate description of photoinduced proton transfer across larger distances or at interfaces, surface hopping with adaptive QM/MM is essential to improve proton diffusion treatment.

Monitoring proton and hydrogen transfers in surface hopping is straightforward. One must keep track of the atom distance to donor and acceptor groups as a function of time (Fig. 5b). Proton and hydrogen transfer can be resolved by additionally checking the CT index (Fig. 5a) or the electronic density.

#### 5.2.2 Proton quantum effects

**Fig. 6:**
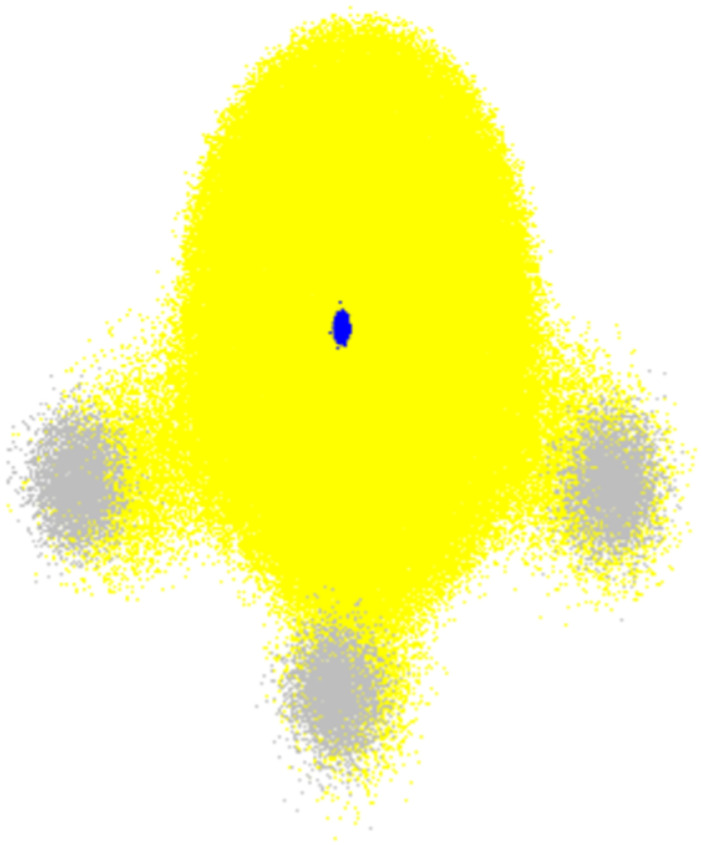
Total electronic density (yellow) and nuclear density of NH_3_ in the ground state. The small blue cloud in the center corresponds to the nitrogen nucleus, while the three grey clouds are the protons. Nuclear densities are plotted with 3000 random configurations sampled from the probability density computed within the harmonic approximation for the normal modes. The electronic density was computed with B3LYP/STO-3G for each nuclear configuration. The plot shows 100 random points for each of these electronic densities. The nuclear density was calculated with Newton-X,^15^ and electronic densities with Gaussian 16.^175^

The main challenge in surface hopping simulations of proton transfer compared to electron transfer is accounting for the proton quantum delocalization, which is illustrated in Fig. 6. While electron delocalization is naturally considered by the electronic structure calculations feeding the dynamics propagation, in conventional surface hopping, the transferring proton (as all other nuclei) is treated as a classical particle.

The proton delocalization problem was recognized in the early 1990s when Hammes-Schiffer and Tully published one of the first applications of surface hopping to proton transfer in solution.^176^ Their surface hopping model extended FSSH to include a proton in the quantum propagation analogously to the electrons. They used the Azzouz–Borgis model^177^ to study the proton transfer reaction in the phenol–amine complex in liquid methyl chloride. This work emphasized the approach's computational feasibility, opening a way to include quantum mechanical phenomena such as tunneling and isotope effects in surface hopping. Later, this model was improved to eliminate classically forbidden transitions to promote consistency between the quantum probabilities and the fraction of trajectories in each adiabatic state.^178^ In another extension, the donor–acceptor vibrational and hydrogen motions were treated quantum mechanically.^179^ Subsequent developments described the solvent as a dissipative bath coupled with a symmetric double well representing the proton transfer reaction.^180^

Nuclear quantum effects can be crucial for describing proton and hydride transfer reaction rates and kinetic isotope effects. The nuclear-electronic orbital (NEO) method has been developed to include such effects.^181^ It presents a computationally practical way to include non-Born–Oppenheimer and quantum effects, such as proton delocalization, zero-point energy, quantized vibrational levels, and tunneling. NEO treats selected nuclei and electrons at the same quantum chemical level. The mixed-nuclear–electronic time-independent Schrodinger equation is solved with molecular orbital techniques. Thus, NEO-methods can generate adiabatic vibronic surfaces on-the-fly during dynamics analogously to conventional electronic structure methods.

Among its different variants, NEO-TDDFT and NEO Multistate-DFT (NEO-MSDFT) can be used to treat proton-transfer reactions, the first in electronically or vibrationally excited states and the second in the ground or vibrationally excited ground state.^181^ A more practical approach, which does not require the calculation of nonadiabatic couplings (as the methods above do), is real-time (RT) NEO-TDDFT. It has been combined with Ehrenfest dynamics to describe ESIPT in *o*-hydroxyaldehyde (*o*-HBA).^182^ Later, the solvent effect was included with a polarizable continuum model.^183^ The use of trajectory surface hopping rather than Ehrenfest dynamics may allow a better description of the branching processes. However, none of the NEO-TDDFT approaches can describe double excitations. More recently, NEO-MSDFT has been combined with both Ehrenfest and surface hopping, allowing a description of hydrogen tunneling in the ground and excited vibronic states of malonaldehyde.^38^ NEO-methods are further discussed in Section 5.3.2.

### 5.3 Proton-coupled electron transfer

Proton-coupled electron transfer (PCET) reactions play a fundamental role in various chemical and biological processes, including energy conversion in photosynthesis and respiration, several enzymatic reactions, and solar energy devices.^184–186^ These reactions are characterized by transferring at least one proton and one electron between the same or different sites, in the same or different directions, *via* stepwise or concerted pathways.^184,185^ PCET occurring after photoexcitation may be referred to by different names depending on the detail of the mechanism. When the electron rearrangement before the proton transfer does not involve a full charge separation, the process is named excited-state PCET.^185^ However, if a full electron is transferred, it is named photoinduced PCET. Most of the time, the two terms are used intermixed. Moreover, photoinduced PCET is also commonly called electron-driven proton transfer (EDPT).^187^ In the following, we will refer to all these processes as photoinduced PCET unless noted otherwise.

Due to their complex regime, accessing the dynamics of PCET reactions is computationally challenging. Different degrees of electron–proton nonadiabaticity emerge from the often strongly coupled dynamics of solute, solvent, transferring protons, and electrons.^185,188^ This issue is directly related to the involvement of several proton–electron vibronic states and the amount of charge distribution during the reaction.^184,185,189^ Moreover, the wide range of time scales involved in the different processes accounting by electron and proton transfers (and solvent response to them) further complicates the description of PCET.^190^ Finally, quantum mechanical effects such as zero point energy, hydrogen tunneling, and transitions between electronic and proton vibrational states may play a critical role and should be considered to describe PCET accurately. This intricate scenario is even more involved for photoinduced PCET, where the reaction can be drastically affected by solvent relaxation from its initial configuration due to changes in the charge distribution of the solute.^185,189^

Over the past decades, a significant effort has been made to develop models for PCET.^185,191,192^ Initially, these approaches focused on thermal-induced processes^184–186,193–195^ with rates derived in the framework of Fermi's golden rule, extending the application domain of Marcus theory. However, rate constants developed for thermal PCET are unsuitable for PCET after a photoexcitation because the former assumes a system in equilibrium before the PCET occurs.^193^ Definitively, this is not the case for photoinduced PCET, as the excitation typically induces an instantaneous change in the charge distribution of the solute, creating a nonequilibrium configuration between the nuclei (solvent and solute) and the new charge distribution. This unique situation requires nonadiabatic dynamics. Details about the theoretical advances in PCET description can be found in various reviews and perspectives.^184–186,193–195^ Here, we focus on applying surface hopping to describe photoinduced PCET.

#### 5.3.1 Proton-coupled electron transfer with surface hopping

**Fig. 7:**
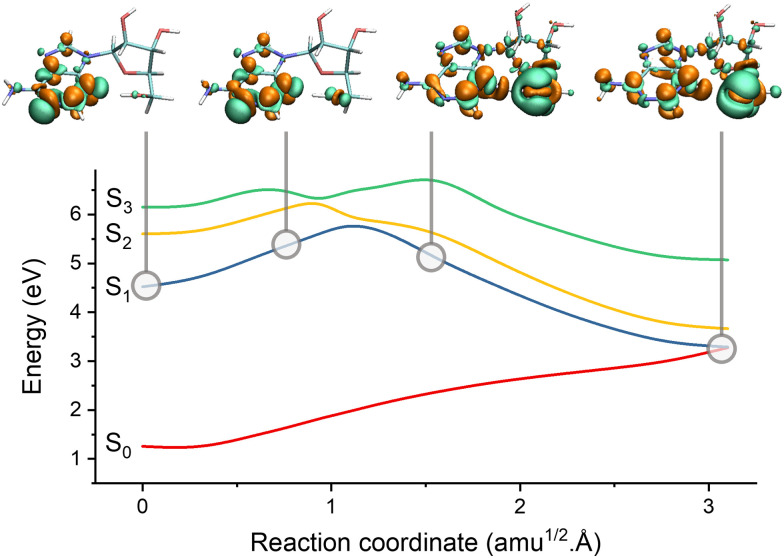
Electron-driven proton transfer (EDPT) in adenosine. The figure shows the potential energies interpolated between the S_1_ minimum and the S_1_/S_0_ EDPT intersection. The snapshots illustrate the electronic density difference between S_1_ and S_0_. Electrons are promoted from green to orange regions. Before the barrier, S_1_ has an n_N_^1^π*^1^ character. It suddenly turns into n_O_^1^σ_NH_^1^ after the barrier due to the proton transfer between the sugar and adenine. Data from ref. 196.

PCET within a classical proton approximation can be trivially simulated with surface hopping. We can illustrate it with recent surface hopping simulations of adenosine in the gas phase.^196^ In this case, adenine is the chromophore, while the sugar group can be considered the environment. Before that work, reaction path simulations for photoexcited adenosine had found that EDPT—with an electron transfer followed by a proton moving from the sugar to adenine—created a conical intersection with the ground state (Fig. 7), which was predicted to be the major internal conversion channel.^197^ However, surface hopping revealed that this pathway plays a minor role in the nonadiabatic deactivation,^196^ and internal conversion is still dominated by ring puckering, like in isolated adenine.

Domcke and co-authors have significantly contributed to EDPT reactions based on classical proton approximation, particularly in DNA nucleobases and small organic molecules.^197–199^ Their surface hopping studies employ small solvent–chromophore clusters interfaced with second-order algebraic diagrammatic construction [ADC(2)] or time-dependent density functional theory (TDDFT). For instance, Pang *et al*.^199^ used Belyaev–Lebedev surface hopping (see Section 2) combined with ADC(2) to investigate how photoexcited pyridine could extract a hydrogen atom from a hydrogen-bonded water molecule within water clusters. More recently, Huang and Domcke^200^ used Belyaev–Lebedev surface hopping with TDDFT to investigate PCET reactions in hydrogen-bonded complexes of trianisoleheptazine, a chromophore related to hydrogen-evolution photocatalysis. Using FSSH and ADC(2), Chaiwongwattana *et al*.^201^ explored the EDPT in adenine monohydrates and showed that EDPT from water to nitrogen is responsible for the ultrafast decay of adenine.

Surface hopping simulations of PCET naturally require including the units involved in the transfer reaction into the QM region. This requirement can be computationally unfeasible for extended systems. As discussed in the context of proton transfer (Section 5.2.2), this problem could, in principle, be alleviated by adopting adaptive QM/MM partitions. Nevertheless, recent results exploring the effect of different QM/MM partitions and force-field types on the surface hopping simulations raised concerns that even adaptive QM/MM partitions may not suffice to describe PCET in extended systems.^96^ In this work, Bondanza *et al*. simulated the EDPT reaction between pyrimidine and water. They observed that adopting a polarized force field has little effect compared to using a conventional force field. Neither could fully predict the same dynamics as simulated with an entire QM cluster. The difference in the results stemmed from the participation of low-lying charge–transfer states involving orbitals delocalized over several water molecules.

Hammes-Schiffer and co-authors recently explored more complex systems, aiming to understand biologically relevant PCET reactions and biomimetic systems. One of these investigations involved photoinduced electron transfer followed by double proton transfer in flavin blue-light (BLUF) photoreceptors.^202,203^ They used Tamm–Dancoff approximation (TDA)/MM dynamics to propagate the system exclusively on S_1_ due to computational costs. After both proton transfers, the ground state acquires a diradical character. At this point, hopping to the ground state is assumed to occur, and spin-flip TDA was used to continue the dynamics in that state. The zero-point energy of the QM subsystem was approximately accounted for by heating it to a high temperature. Despite the strong approximations, these investigations provide the foundations for a comprehensive protocol to account for the various mechanistic pathways and timescales involved in complex PCET reactions.

All research surveyed in this subsection considered classical protons in surface hopping propagation. Since the transfer reactions were ultrafast in all these studies, a classical transfer should dominate over tunneling,^204^ and we expect these results to be qualitatively correct.

#### 5.3.2 Nuclear quantum effects in PCET

As discussed in Section 5.2.2, Hammes-Schiffer and Tully proposed a surface hopping formulation where transferring protons, like electrons, are included in the quantum-mechanical treatment.^176^ This formulation is still the basis for dealing with proton quantum effects during proton transfer and PCET in surface hopping. Extensions of this formulation were further developed to be applied to photoinduced PCET^205^ (and vibrational energy transfer, as surveyed in Section 6.2.1). The first models used surface hopping in conjunction with Langevin equations of motion to simulate the nonadiabatic dynamics on the electron–protonvibronic energy surfaces after photoexcitation.^205,206^ The PCET dynamics depend on the solvent coordinates, which are either described as single collective^205^ or multiple scalar^206^ solvent coordinates using dielectric continuum theory. Further development of these two-dimensional model systems included the solvent explicitly.^207^ These works illustrate how the solvent dynamics can be tuned by altering the solute and solvent properties and suggest that implicit approaches may be suitable for investigating a variety of photoinduced PCET.^208,209^

Photoinduced PCET in extended systems was further investigated using surface hopping with QM/MM. Goyal, Hammes-Schiffer, and co-authors explored the photoinduced PCET in a hydrogen-bonded phenol-amine complex in solution.^208,209^ They used a reparametrized semi-empirical implementation of the floating occupation molecular orbital complete active space configuration interaction (FOMO-CASCI)^210,211^ to describe the solute. The quantized proton was represented as a quantum mechanical wave function computed with the Fourier grid Hamiltonian method. These works (reviewed in ref. 185, 193 and 195) represent significant advances in the nonadiabatic formulation of photoinduced PCET and mark the inclusion of quantum chemical treatment for the transferring proton using surface hopping in realistic systems.

Methods such as path integral,^212^ multiconfigurational time-dependent Hartree (MCTDH),^213^ quasi-diabatic formalism,^194^ and quantum-classical Liouville^202^ have also been proposed to include nuclear quantum effects. (Brown and Shakid discussed the recent progress in approximate quantum dynamics methods to investigate PCET.^214^) For instance, the quantum-classical Liouville equation (QCLE) may provide a solid semiclassical framework to overcome many limitations in conventional surface hopping. In surface hopping formulations based on this approach, the numerical solution of the environment's degrees of freedom is treated classically, while electrons and transferring protons are treated quantum mechanically.^212^ QCLE thoroughly describes the nonadiabatic transitions between proton–electron vibronic states, accounting for decoherence effects and providing exact rate constants.^213^ However, the algorithms developed for solving the QCLE equations suffer from numerical and convergence instabilities, requiring a large ensemble of trajectories to obtain satisfactory results. Liu and Hanna^215^ addressed such a problem by proposing a new surface hopping algorithm that solves a deterministic set of coupled first-order differential equations for the bath and subsystem and constructs observables for time-dependent coordinates from there. The model was applied to photoinduced electron transfer, opening new horizons for studying PCET reactions.

Recently, Coffman *et al*.^216^ proposed using the QCLE embedded into a classical master equation (CME) for simulating PCET reactions in the context of voltammetry curves. (A comprehensive overview of electrochemical PCET can be found in ref. 217.) The final QCLE-CME equation can be solved by an algorithm combining diffusion of the reactant and product in solution and surface hopping between electronic states. It uses a generalized Anderson–Holstein Hamiltonian model for PCET, including quantized proton coordinates, while the coordinates influencing electronic motion are treated classically. Their findings reveal a qualitatively agreement upon the addition of nuclear effects through the proton coordinate. Overall, these results suggest that the current–voltage curves are insufficient to determine if the PCET reaction occurs or not. Combining current–voltage simulation methods with more accurate potential energy surfaces would lead to further mechanistic insights. This work opens the doors for the investigation of electrochemical PCET reactions and more complex PCET scenarios.

As introduced in Section 5.2.2, NEO methods are suitable alternatives for incorporating nuclear quantum effects in PT reactions, as they treat all the electrons and transferring-protons quantum mechanically.^218^ The application of various NEO methods for direct dynamics simulations on electron–proton vibronic states was recently discussed in ref. 181. Although NEO methods have been designed to be suitable for PCET reactions, up to now, nonadiabatic dynamics simulations using this approach have been restricted to PT reactions (see Section 5.2.2).

## 6. Energy transfer

### 6.1 Electronic energy transfer

Electronic excitation energy transfer is vital for many biological processes (such as photosynthesis^84,219^) and technological applications (for example, photovoltaics cells^220,221^ and photochemical switches^222,223^). Electronic energy transfer involves the transfer of the energy absorbed by a chromophore (the donor) to a nearby acceptor, which is then promoted to the excited state.^224^

**Fig. 8:**
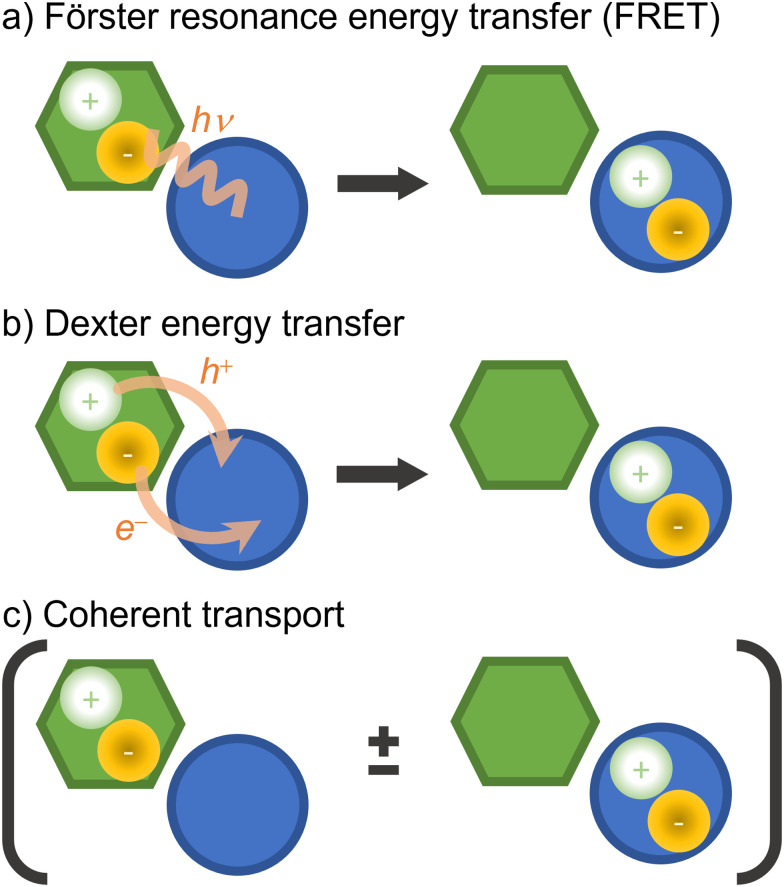
Types of electronic energy transport. In the schemes, the hexagon and the circle represent different chromophores. (a) FRET: a localized exciton in one chromophore is transferred to the other through energy resonance (indicated by the virtual photon, *hν*). (b) Dexter energy transfer: a localized exciton is transferred at short distances *via* electron exchange between the chromophores (indicated by the electron, e^−^, and electron–hole, h^+^, transfers). (c) Coherent transport: an entangled quantum exciton is formed over the chromophores. The transport occurs when the electronic wave function collapses to a localized excitation. Although the diagrams illustrate neighbor chromophores, they can be far from each other in FRET and coherent transport.

There are a few distinct types of electronic energy transfer, each with different mechanisms and properties (Fig. 8).^225^ Förster resonance energy transfer (FRET) involves electronic energy transfer from one molecule to another, typically over longer distances. It is mediated by a Coulomb coupling between the donor and acceptor molecules. Dexter transport occurs *via* electron exchange between neighboring molecules (which can also be seen as a transfer of an electron and a hole from a donor to an acceptor) and is typically limited to short distances. FRET and Dexter are characterized by an incoherent transport (at a specific time, only a single site is excited) well suited for hopping algorithms. Coherent transport, conversely, is a quantum mechanical process where excitons propagate through the material as a wave *via* entangled excitations at different sites. The transport only occurs after the wave function collapses into the excitation of a specific site.^226^ We can generally and schematically represent electronic energy transport through the reaction A* + B → A + B*, without distinguishing the underlying mechanism.

An outstanding electronic energy transfer example is multichromophoric DNA, whose excited-state lifetime can range from 10 to 100 ps due to excitonic and delocalized charge–transfer processes.^227^ For an overview of the quantum-chemical methods applied to electronic energy transfer in the context of light-harvesting systems, we refer the reader to ref. 52 and 84. Recent developments in the simulations of exciton transport in the FRET, coherent, and intermediary regimes are discussed in ref. 228.

#### 6.1.1 Electronic energy transfer with surface hopping

In a multichromophoric system, the migration of excitation energy between chromophores implies a dramatic change in the electronic wave function, involving multiple potential energy surfaces so that nonadiabatic dynamics is required. The size of these extended systems, where all units should be treated at a quantum mechanical level, poses a natural bottleneck for these simulations. From the three types of electronic energy transfer illustrated in Fig. 8, FRET is the most amenable for surface hopping. In FRET, the localized exciton jumps incoherently between chromophores. Thus, the quantum mechanical description may be restricted to single chromophores with classical interactions between them.^229^ This simplification allows incorporating fragment approaches in surface hopping, as discussed in this section later. FRET simulations often use hopping algorithms based on Frenkel excitonic Hamiltonians to propagate exciton diffusion.^229^ Those hopping algorithms should not be mistaken for surface hopping between adiabatic surfaces. The excitation jumps from one site to another, but the nuclear dynamics may even be restricted to a single adiabatic surface.

Both Dexter and coherent energy transfer require quantum treatment of multiple chromophores. Dexter transfer is somewhat simple to simulate with surface hopping because it is restricted to first-neighbor chromophores.^230^ Coherent transfer simulations are significantly more complex due to the required QM region size, the electronic densities' complexity, and the decoherence treatment. Indeed, this last point is one of the conceptual weaknesses of surface hopping, which does not distinguish between decoherence and wave function collapse.^231^

Supposing that the entire system can be treated at a quantum mechanical level, surface hopping will simulate the energy transfer process without any *a priori* definition of the mechanism. It is a matter for the post-simulation analysis to classify the transfer as Dexter, FRET, coherent, or any intermediary case. Moreover, surface hopping automatically accounts for couplings between electrons and nuclear vibrations. These features once more illustrate the discovery power of surface hopping, which we had already highlighted in the context of reaction coordinates (Section 5.1.1).

**Fig. 9:**
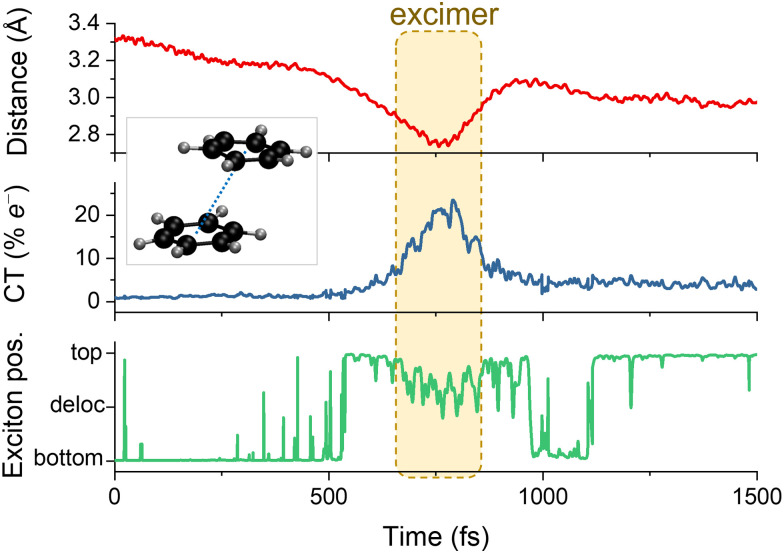
Characterization of the electronic energy transfer processes during a single surface hopping trajectory of benzene dimer in parallel stacked conformation. Initially, the bottom benzene is photoexcited, making it the chromophore and the top molecule, the environment. The upper graph shows the distance between the monomers' centers of masses. The middle one gives the charge transfer (CT) index. The bottom graph illustrates the exciton position, which can be localized in each monomer or delocalized over them. A short-lived excimer (an excited bound dimer without a corresponding bound ground state) is formed when the CT character mixes in the wave function. Data from ref. 160.

The case illustrated in Fig. 9, the exciton transfer in a benzene dimer, is an example of a brute force approach, where the entire system is treated at the QM level.^160^ However, the brute force approach is rarely the best option for realistic extended systems unless we accept a dramatic reduction in the electronic structure accuracy.

Because of the physical separation between chromophores, localized states (the excitons) are examples of diabatic states whose interaction matrix elements determine the transition probabilities.^232^ From the computational point of view, the diabatic representation based on the localization of charge and excitation is a natural choice for analyzing electronic energy transfer. However, most computational methods approximate the energies and properties using adiabatic eigenstates. There are several good reasons to switch to a (quasi) diabatic basis. An efficient strategy to deal with multichromophoric systems is to use a fragment approach (aka divide-and-conquer), which splits the system into sub-units and evaluates the state energies of each one and the interaction between them (see EXASH in Section 4.2). By doing so, the strategy is intrinsically diabatic.^232^

A successful divide-and-conquer technique to study electronic energy transfer in multichromophoric systems is the Frenkel exciton model. In this model, a linear combination of localized excitations represents the excited states of the multichromophoric system.^98^ The system is described using the transition energies of localized excitations and the couplings between them. This information can be inserted in a pure electronic model Hamiltonian to obtain electronic energy transfer rates from various approaches^233^ or used to perform full-dimensional nonadiabatic simulation of multichromophoric systems.^98^ To this end, implementations conciliating the Frenkel exciton model and trajectory-based mixed quantum-classical dynamics have been proposed.^52,98,234,235^

Sisto and co-workers used the exciton model combined with surface hopping to investigate the excitation energy transport in the light-harvesting complex II.^235^ In their model, the chromophores are coupled *via* Coulomb dipole–dipole interactions, and electronic wave functions are based on the excitonic eigenstates of individual chromophores. Menger and co-authors extended this model by introducing a QM/MM-like electrostatic embedding scheme, which allowed the investigation of chemically bonded chromophores.^234^ Surface hopping based on TDDFT/MM was also used to model the nonadiabatic dynamics of stacked adenine tetramer in a single-strained DNA.^236^ In their model, the four adenine bases were treated at the QM level, while the remaining system was treated using MM. In both models, the excited state dynamics is initiated with one chromophore getting excited close to the absorption band.

Although TDDFT is computationally affordable, it suffers from inherent problems associated with its single reference character.^27^ Semi-empirical approaches like the configuration interaction based on the orthogonalization method 2 (OM2/MRCI)^237^ or the floating occupation molecular orbitals-configuration interaction method (FOMO-CI)^210^ offer computationally affordable multireference electronic structure options.^238^ A downside of using semi-empirical methods is the need for reparameterization in most cases.

Wohlgemuth and Mitrić combined electrostatic embedding OM2/MRCI/MM with multichromophoric field-induced surface hopping (McFISH) to describe the exciton energy transport in DNA in the framework of the Frenkel exciton model.^227^ The QM part was defined by multiple subsets containing stacked base pairs and treated with OM2/MRCI, while the DNA backbone and other nucleobases, water, and ions were treated *via* a classical force field. Each QM subsystem was individually embedded in the charge field of the other monomers and the solvent. The coupling between them was determined through the transition dipole moments. This model was able to predict the formation of long-lived delocalized excitonic and charge transfer states as well as ultrafast decay of excited states in double-strained DNA. This approach was later used to investigate ultrafast energy transfer in squaraine heterotriad.^239^

Sangiogo Gil *et al*. recently presented an excitonic model which combines the Frenkel exciton model and surface hopping to investigate the electronic energy transfer in multichromophoric systems.^98^ In this model, the energies and couplings between the chromophores are evaluated using FOMO-CI. The Coulomb exciton coupling is computed either exactly (by using the semi-empirical approximation) or approximated by using transition atomic charges. For a minimal multichromophoric model consisting of *trans*-azobenzene-2*S*-phane molecule, both approaches provide a good agreement with a full-QM procedure. Subsequently, they extended their excitonic model to simulate a self-assembled monolayer of an azobenzene derivative on a gold surface, obtaining a good agreement with experimental results.^240^ Their methodology is not restricted to FOMO-CI and can be adapted to other electronic structure methods. In Newton-X,^15^ for example, it is implemented for FOMO-CI and TDDFT.

The weak link of the previous fragment approaches is that quantum delocalization is restricted to the fragment. In many cases, one may require a flexible methodology to accommodate delocalized excitons. Besides that, thermal vibrations coupled with the electronic interactions and the effect of electronic interactions on the nuclear motion (*back reactions*) needed to be considered. Giannini and co-workers introduced the FE-SH,^103^ which addresses these issues (see Section 4.2). The methodology considers the thermal fluctuations of excitonic couplings and site energies beyond the harmonic approximation. From this study, they proposed a way to rationally improve exciton transport in organic optoelectronic materials. FE-SH was used to investigate the exciton diffusion in molecular organic crystals such as non-fullerene acceptors.^103^ Extensions of the model would, ideally, be able to deal with exciton dissociation to interfacial charge–transfer states, charge separation, and recombination, which are underlying principles of organic solar cells. Indeed, a recent extension, the X-SH model (also discussed in Section 5.1.1), allows the simulation of localized exciton dissociation to charge–transfer states. It was applied in a model organic interface and is a promising method to simulate organic optoelectronic materials at the nanoscale.

In practical terms, monitoring electronic energy transfer can be done post-processing surface hopping results. For instance, checking 1-electron density descriptors such as position descriptor (Fig. 5c),^161^ whose values indicate the monomer the exciton is located or whether it is delocalized.

### 6.2 Vibronic and vibrational energy transfers

**Fig. 10:**
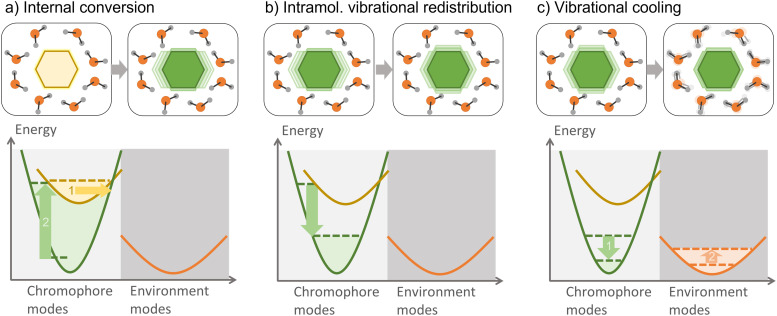
Main vibronic and vibrational energy transfer processes. (a) Internal conversion: an electronically excited chromophore returns to the ground state without emitting radiation. The excess energy is transferred to specific vibrational modes of the chromophore in the electronic ground state. (b) Intramolecular vibrational energy redistribution (IVR): vibrational energy dissipates to other vibrational modes of the chromophore. (c) Vibrational cooling: the hot chromophore dissipates its vibrational energy to the environment's rotational, vibrational, and translational modes.

Vibrational energy transfer is ubiquitous when dealing with non-equilibrated systems, such as those resulting from an internal conversion. After the photoexcitation of a chromophore in an environment, vibrational relaxation—one of the several relaxation processes which can take place—is usually split into internal vibrational redistribution (IVR) and vibrational cooling (Fig. 10). The interplay between these two processes is helpful in numerous applications, such as heating transfer in biological systems,^241^ molecular heaters,^242^ photothermal therapy,^243^ and thermal conduction in molecular junctions.^244^ In large molecules, several vibrational modes are optically active; therefore, vibronic states should be considered.

An initially localized vibrational excitation tends to spread over the molecular framework, thermalizing the vibrational modes. This process is referred to as intramolecular vibrational redistribution (IVR).^245,246^ IVR happens in the excited state after photoexcitation, which lifts the ground-state wave function to an excited state, where it is no longer an eigenvector of the Hamiltonian, yielding a wave packet evolving with time among the vibrational modes. IVR also happens after internal conversion to a lower electronic state, converting electronic energy into vibrational energy and creating a thermally unequilibrated molecular system.^247^ The theory of the internal temperature of entirely isolated systems is discussed in ref. 248.

When the hot chromophore is not isolated, it can equilibrate with its surroundings through vibrational cooling.^249^ Thus, vibrational cooling is an energy flow from the chromophore to the immediate environment, from where the heat dissipates to the bulk. As for IVR, vibrational cooling can occur in the S_1_ or S_0_ electronic state. Vibrational cooling tends to occur in the ground state if the chromophore is not covalently bounded to the environment and the internal conversion is ultrafast. Moreover, IVR and vibrational cooling are typically treated as sequential processes, but this separation is sometimes questionable, as they can occur on similar time scales.^247^ When vibrational cooling happens, the chromophore heating reduces (compared to the heating of an isolated chromophore) because of the leaking into the environment.

#### 6.2.1 Vibronic and vibrational energy transfer in surface hopping

In all previous sections discussing charge and electronic energy transfer, we have pointed out that the main challenge for surface hopping was including the relevant parts of extended systems in the QM region. This is not the case with vibrational energy transfer, which is reasonably well described by force fields and model Hamiltonians. If fact, historically, many of the first applications of surface hopping used such approximations to investigate electronic energy transfer into vibrational, rotational, and translational modes of small molecules to simple environments, like atom-diatomic or diatomic in rare gas matrices.^250,251^

Bastida, Fernandez-Alberti, and co-authors have pioneered these applications by including a few nuclear degrees into the quantum partition of surface hopping.^252–254^ Their method, named molecular dynamics with quantum transitions (MDQT), is an adaptation of the Hammes-Schiffer and Tully approach^176^ (which we discussed in Section 5.2.2) and can be used to study ground-state vibrational energy relaxation with or without photoexcitation. Their extensive repertoire of case studies includes from I_2_ photodissociation within an Ar matrix^252^ to the challenging treatment of HOD vibrations in liquid H_2_O.^254^

Surface hopping has also been applied to study vibrational energy relaxation in the condensed phase using model Hamiltonians.^255,256^ A common choice is to employ the spin-boson Hamiltonian, which couples a two-state system to a harmonic bath. A semiclassical surface-hopping propagator has been developed and applied to the study of the vibrational relaxation of Br_2_ in Ar.^257^ This propagator is a useful mathematical tool that can be employed in expressions for the probability of transitions between quantum states of molecules in condensed phases.

Surface hopping based on QM/MM can cope with internal conversion, IVR, and vibrational cooling on equal footing, independent of their time scales and even when they compete within the same time scales.^247^ By monitoring the time dependence of the kinetic energies of the solute and solvent molecules (Fig. 5f), it is possible to study the heating and cooling of both solvent and solute.^258^

Our group has recently applied QM/MM surface hopping followed by kinetic energy analysis to determine the energy-transfer time constants of cytosine, a prototypical organic chromophore, in three different environments, argon matrix, benzene, and water.^88^ Internal conversion heats cytosine in the sub-picosecond scale (independently of the environment) and cools it down within 25 ps in argon, 4 ps in benzene, and 1.3 ps in water. These values were determined with a kinetic model connecting the chromophore heating rate with the decay rates of the excited states’ populations and the chromophore cooling rate with the scaled kinetic energy difference between solute and solvent.

The relevant nuclear motions involved in the energy transfer can be identified by projecting velocities onto the normal modes (Fig. 5e).^259^ Fernandez-Alberti, Tretiak, and co-authors have employed this approach to analyze vibronic energy transfer and IVR in large chromophores and an acceptor–donor system.^259,260^ They identified that immediately before internal conversion, the dynamics involved a small number of normal modes, all with considerable overlap with the nonadiabatic coupling vector. After internal conversion, the number of active normal modes increased, characterizing the starting of IVR.

Normal-mode projection could also be applied to analyze vibrational cooling. However, it stumbles on determining the normal modes of extensive systems. Huix-Rotllant and Ferré proposed a promising approach that can lift this restriction.^261^ They implemented a new electrostatic embedding QM/MM method based on new charge operators employing atom-centered grids, which scales linearly with the MM subsystem size due to the analytic energy, gradient, and Hessian matrix.

#### 6.2.2 Zero-point energy leakage

As with any method based on classical dynamics of the nuclei, surface hopping may present artifacts due to zero-point energy (ZPE) leakage.^262^ This problem occurs because classical dynamics do not enforce zero-point energy constraints. Thus, high-frequency vibrational degrees of freedom (such as a CH stretching mode) may lose energy toward low-frequency modes, ending with less than the zero-point energy, the minimum amount it must have. This spurious energy transfer may artificially trigger rotational and translational motions.

Surface hopping simulations of chromophores surrounded by unbound environment molecules are particularly threatened by ZPE leakage due to their weak interaction. In this case, the leaking can cause the dissociation of an otherwise bound system. Such an effect happens, for instance, to the ground-state dynamics of water dimers.^262^

ZPE leakage has been under the radar of dynamics developers for decades, and many solutions have been offered.^263,264^ Nevertheless, most of them require knowing Hessian matrices during dynamics, which is particularly troublesome and costly when propagating with on-the-fly strategies, as usually done in surface hopping.

The local-pair ZPE correction is a new approach that does not require Hessian matrices, thus being tailored for on-the-fly propagation.^30^ It monitors the mean kinetic energy of pairs of atoms vibrating at high frequencies. Whenever this mean value drops below some pre-established threshold, the velocities are rescaled to replenish the missing kinetic energy. Total energy conservation is enforced by removing equivalent kinetic energy from all other pairs of atoms (not necessarily bound). All velocity rescalings are done in a way that conserves linear and angular momentum.

## 7. Future directions on surface hopping modeling of charge and energy transfer

Charge and electronic energy transfer simulations face the ever-present challenge of treating extensive supramolecular systems at a quantum level. (Consider, for example, the electron transfer between a photoexcited chromophore and a surface or an exciton jumping between chromophores in an organic crystal).

Surface hopping methods based on fragment Frenkel-exciton models are good strategies for electronic energy transfer.^98^ Their shortcoming is that those models assume electronic excitations are localized on weakly interacting chromophores. Therefore, strong interactions (or reactions) between the units, including charge delocalization and charge transfer, are still challenging to describe. A strategy would be enlarging the QM region of each unit, but this may not be computationally affordable in many cases.

For a more complete picture, the most promising surface hopping algorithms are based on fragment approaches. X-SH, for instance, can describe charge and energy transfer and can, in principle, be extended to account for charge delocalization, electron–phonon couplings, recombination to the electronic ground state, the influence of interface geometry, and static disorder.^104^ However, these fragment–orbital-based methods are highly parametrized and still restricted to one-particle electronic wave functions. Divide-and-conquer fragment approaches like EXASH,^98^ although more computationally demanding, may overcome those limitations if they are generalized to also deal with charge transport.

In the case of proton transfer, proton-coupled electron transfer, and some instances of vibrational energy transfer, nuclear quantum effects add a new challenge to the previous ones. Surface hopping can tackle these effects, either including the proton in the FSSH time-dependent wave function or employing vibronic surfaces, where the proton wave function is considered during the electronic structure calculations. The latter option seems to get more traction after NEO methods started to appear in standard quantum-chemistry programs like GAMESS and Q-Chem.^181^

NEO methods can be combined with trajectory surface hopping or other dynamics methods to describe PT and PCET. Using NEO with surface hopping follows the same cost/benefit logic of a conventional electronic structure method. Linear-response NEO-TDDFT is the most computationally affordable option. However, the description of doubly excited states or radical systems will require NEO wave function multiconfigurational methods. Ideally, simulations of nonadiabatic processes in extended systems involving proton transfer should count on surface hopping with adaptive QM/MM and NEO-based methods. Such an algorithmic combination could deliver high-quality results at affordable computational costs.

Supposing we have the methods for performing dynamics, there are a few challenges to address in analyzing the results.

Concerning charge transfer, different descriptors have been proposed to characterize it during or after the simulations. However, they do not necessarily deliver the same results, and depending on the quantity used to define an electron-transfer event, one can obtain more or less charge delocalization. A general, transferrable, and black-box protocol for estimating rates is required. A potential candidate is the CT index based on the transition density matrix. This approach has been used to assign charge transfer during surface hopping before,^160^ but its use to compute electron-transfer rates was not explored. Nevertheless, even if we get such rates, the results may depend on the decoherence-correction approach adopted^157^ when using FSSH-based methods. This critical point deserves more investigation to establish a precise simulation protocol.

Descriptors based on the transition density matrix are also relevant for characterizing electronic energy transfer. Nevertheless, they do not allow assigning specific mechanisms, like resolving Dexter from FRET. Therefore, this is another topic requiring development attention.

In the case of vibrational energy transfer, identifying normal modes responsible for the transfer is the key to understanding the energy flows between a chromophore and the environment. However, normal mode analysis is still unpractical for extensive systems. Recently developed methods to calculate linear-scaling Hessian matrices of MM regions^261^ are promising approaches for such analysis in QM/MM setups.

## 8. Conclusions

Photoexcitation of a chromophore creates an unequilibrated molecular system, triggering a cascade of nonadiabatic processes. Solvents, biomolecular structures, crystals, or surfaces surrounding the chromophore may change the nature of these processes, characterizing an active environment. Such an active environment impacts the chromophore's dynamics by modifying the potential energy landscape and enabling energy and charge flows, which are impossible when the chromophore is isolated.

This Perspective surveyed the latest theoretical and computational developments in the simulations of nonadiabatic processes in active environments based on surface hopping molecular dynamics, focusing on energy and charge transfers. Naturally, dealing with extended systems, including chromophore and environment, is the greatest computational challenge of these simulations, especially when it is indispensable to account for quantum effects in the environment, in nuclear degrees of the chromophore, or both. Computational chemists have developed an arsenal of techniques to deal with various situations.

Surface hopping has repeatedly revealed extreme algorithmic plasticity to incorporate these techniques and deliver powerful insights about nonadiabatic dynamics. As a successful application of surface hopping is strongly dependent on the electronic structure level, developing and implementing more accurate methods also imply improvements in the surface hopping results. We still have many challenges to face regarding the accuracy, size, and time scales. However, most of them already have solid potential solutions. Maybe the main difficulty is that the software programs encoding many of these approaches are not generally available to the entire community.

## Author contributions

JMT, MTC, EV, SAM, and MB: conceptualization and investigation. EV, SAM, and MB: fund acquisition. MB: project administration and software. JMT and MB: supervision. MB: visualization. JMT, MTC, EV, SAM: writing – original draft. JMT and MB: writing – review & editing.

## Conflicts of interest

There are no conflicts to declare.

## Supplementary Material
